# Notopterol Attenuates Estrogen Deficiency-Induced Osteoporosis *via* Repressing RANKL Signaling and Reactive Oxygen Species

**DOI:** 10.3389/fphar.2021.664836

**Published:** 2021-06-03

**Authors:** Delong Chen, Qingqing Wang, Ying Li, Ping Sun, Vincent Kuek, Jinbo Yuan, Junzheng Yang, Longfei Wen, Haibin Wang, Jiake Xu, Peng Chen

**Affiliations:** ^1^ Department of Orthopaedic Surgery, Clifford Hospital, Jinan University, Guangzhou, China; ^2^ School of Biomedical Sciences, University of Western Australia, Perth, WA, Australia; ^3^ Department of Orthopaedic Surgery, Sir Run Run Shaw Hospital, Zhejiang University School of Medicine, Hangzhou, China; ^4^ Department of Orthopaedic Surgery, Third Affiliated Hospital, Guangzhou University of Chinese Medicine, Guangzhou, China; ^5^ Department of Endocrinology, First Affiliated Hospital, Guangdong Pharmaceutical University, Guangzhou, China; ^6^ Guangzhou University of Chinese Medicine, Guangzhou, China; ^7^ Department of Orthopaedic Surgery, First Affiliated Hospital, Guangzhou University of Chinese Medicine, Guangzhou, China

**Keywords:** notopterol, osteoclastogenesis, osteoporosis, nfatc1, ROS

## Abstract

Integrity of the skeleton is sustained through the balanced activities of osteoblasts and osteoclasts in bone remodeling unit. The balance can be disrupted by excessive osteoclasts activation commonly seen in osteoporosis. Notopterol (NOT) is a main component of Notopterygium incisum which exerts a wide spectrum effect on biomedical pharmacology. In our study, we found NOT serves as an inhibitor in regulating RANKL-activated osteoclasts formation and bone resorption function by calculating tartrate resistant acid phosphatase (TRAcP) staining and hydroxyapatite resorption assays. Furthermore, RANKL-mediated signaling pathways including MAPK, NF-κB and calcium ossification were hampered, whereas ROS scavenging enzymes in Nrf2/Keap1/ARE signaling pathways were promoted by NOT. In addition, the activation of the essential transcription factor NFATc1 in RANKL-mediated osteoclastogenesis was almost totally suppressed by NOT. What is more, NOT diminished the loss of bone mass in preclinical model of OVX mice by blocking osteoclastogenesis determined by bone histomorphometry, TRAcP staining and H&E staining. Conclusively, our findings demonstrated that NOT could arrest osteoclastogenesis and bone resorptive activity by attenuating RANKL-mediated MAPK, NF-κB, calcium and NFATc1 signaling transduction pathways and enhancing ROS scavenging enzymes in Nrf2/Keap1/ARE pathways *in vitro*, and prohibit bone loss induced by OVX *in vivo*. Taken together, NOT may be identified to be a natural and novel treatment for osteolytic diseases.

## Introduction

Bone is a metabolically active organ that persistently remodeling and undergoes regeneration throughout the whole life (Yin et al., 2005). This continuous remodeling is concerned with the removal of mineralized bone through osteoclasts and subsequent the formation of bone matrix by osteoblasts (Hadjidakis and Androulakis., 2006; Chen et al., 2018). Physiologically, these two activities must be closely coupled both in quantity as well as in time and space. Maintaining the correct coupling play a crucial part in keeping the health homeostasis of bone mass and bone architecture (Samsa et al., 2016). Under pathological conditions, architectural destroy of the skeleton and bone mass loss occur when the bone coupling activity is disturbed owning to the dominant activities of osteoclasts in osteoporosis or during old age (Knowles, 2015; Sun et al., 2020).

Osteoclasts are bone resorptive cells with highly specialized motile migratory, originated from the osteoclast precursor cell (Chen et al., 2013; Cui et al., 2018).^7, 8^ These cells possess marked phenotypic and morphological traits that are frequently utilized to identify them, such as multinuclearity and expression of various of osteoclast markers, including TRAcP, *calcitonin* receptor, cathepsin K (CTSK) and vacuolar-type H + ATPase V0 subunit d2 (V-ATPase-d2) (Tanaka et al., 2013; Wu et al., 2014; Clarke et al., 2015). Macrophage colony-stimulating factor (M-CSF) and receptor activator of nuclear factor-κB (NF-κB) ligand (RANKL) are two independent cytokines for survival, proliferation, and differentiation of bone marrow monocytes *in vitro* (Kikuta et al., 2013; Tsubaki et al., 2014). RANKL and RANK binding promotes recruitment of tumor necrosis factor receptor-associated factors (TRAFs) to the RANK cytoplasmic domain, thereby giving rise to the stimulation of distinct signaling cascades mediated by mitogen-activated protein kinase (MAPK), NF-κB and calcium oscillation (Nakamura et al., 2003; Callaway and jiang., 2015; Li et al., 2016; Lee et al., 2018). Of great importance, RANKL-activated nuclear factor of activated T cells 1 (NFATc1) signaling pathway also plays a pivotal switch role in modulating osteoclasts precursor cells terminal differentiation (Kim et al., 2014). Therefore, deficiency of RANKL-induced cellular and molecular function can be practical for the prevention and management of osteoclast-associated disease.

Notopterol, one type of furanocoumarin, is an active compound isolated from Notopterygium incisum (also termed as Qianghuo in Chinese herbal medicine) with a wide spectrum effect on biomedical pharmacology (Wu et al., 2017). NOT possesses the analgesic and anticancer activities (Huang et al., 2019). However, the impact of NOT on RANKL-activated osteoclastogenesis and bone resorptive function is still elusive. Here, our data indicated that NOT blocked RANKL-induced osteoclast differentiation as well as bone resorptive activity in dose-dependent and time-dependent ways. Interestingly, NOT served as an inhibitor in RANKL-activated reactive oxygen species (ROS) and NFATc1 signal transduction pathways. What is more, ovariectomized (OVX) mouse model was introduced to examine whether NOT could be a protective agent in bone loss resulted from estrogen deficiency mice. Consistent with our results from *in vitro* experiments, NOT remarkably repressed bone loss caused by OVX and attenuated the amount of osteoclast number. These data definitely confirmed that NOT suppressed osteoclast precursor cells differentiation and bone resorptive function *via* blocking RANKL-induced MAPK, NF-κB, calcium as well as NFATc1 signaling pathways and promoting the expression of ROS scavenging enzymes in NF-E2-related factor 2 (Nrf2)/kelch-like ECH-associated protein-1 (Keap1)/antioxidant response element (ARE) signaling pathways. These data also suggested that Notopterol is a novel and potential inhibitor in managing osteolytic bone disease.

## Materials and Methods

### Materials and Chemical Reagents

**FIGURE 1:**
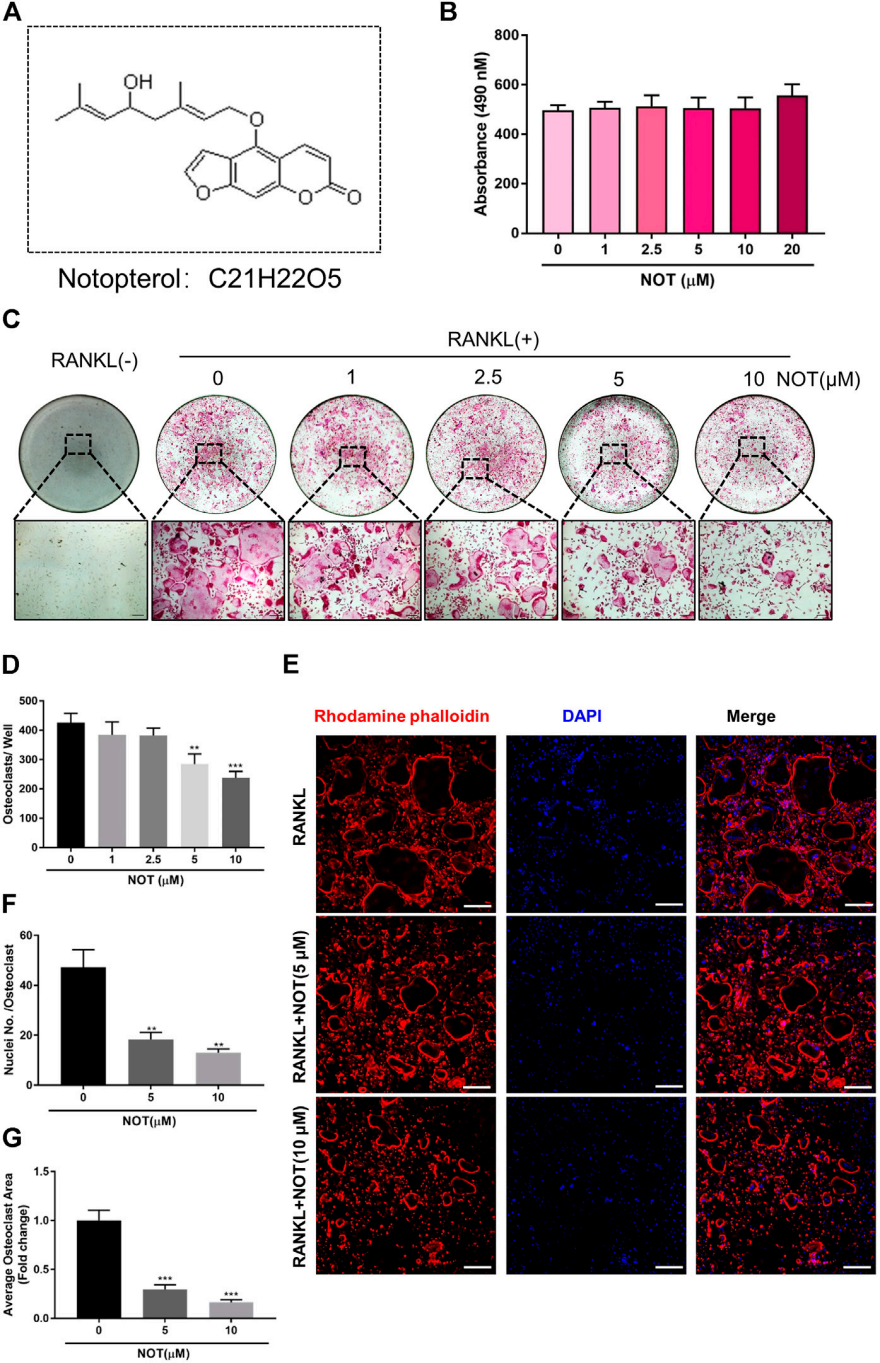
Notopterol inhibits RANKL-induced osteoclastogenesis *in vitro*. **(A)** Chemical structure of Notopterol. **(B)** Effect of indicated concentrations Notopterol on viability of BMMs as measured by MTS assay. **(C)** Representative light microscope images of RANKL-induced osteoclast formation treated with indicated concentrations of Notopterol for 5 days (Scale bar = 200 μm). **(D)** Quantification of TRAcP-positive multinucleated cells (nuclei >3) treated with indicated concentrations of Notopterol. **(E)** Representative confocal images of mature osteoclasts stained for F-actin belts and nuclei (Scale bar = 200 μm). **(F)** Quantification of the average number of nuclei involved in TRAcP-positive multinucleated cells. **(G)** Quantification of the area of F-actin belts occupied by osteoclasts per field. “−” means RANKL untreated; “+” means RANKL treated. Data are presented as mean ± SEM. All *in vitro* experiments were repeated three times with similar results. **p < 0.01, ***p < 0.001 relative to RANKL-induced controls.

Notopterol with purity >98% (Figure 1A) was purchased from Chengdu Herb purify CO, LTD (Chengdu, China), prepared to be a stock solution (100 mM) with dimethyl sulfoxide (DMSO) and then diluted to working solution with phosphate buffer saline (PBS). Alpha modified minimal essential medium (α-MEM), fetal bovine serum (FBS) and penicillin/streptomycin were obtained from Thermo Fisher Scientific (Scoresby, Australia). Primary antibodies for IκB-α (Cat# L2010), p-ERK1/2(Cat# D1117), ERK1/2(Cat# E1717), NFATc1(Cat# G3014), Cathepsin K (Cat# C0810) and β-actin (Cat# J0914) were purchased from Santa Cruz Biotechnology (San Jose, CA, United States). Primary antibodies targeting phosphorylated (p) −JNK1/2 (Cat# 9252S), JNK1/2 (Cat# 9252L), p-P38 (Cat# 4511L), P38 (Cat# 9212L), p-P65 (Cat# 3033S), P65 (Cat# 8242S), p-PLC gamma2 (Cat# 3871S), PLC gamma2 (Cat# 3872S), MMP9 (Cat# 13667S), c-Fos (Cat# 2250S), HO-1 (Cat# 70081S) and catalase (Cat# 12980S) were acquired from Cell Signaling Technology (Beverly, MA, United States). M-CSF and RANKL were used in these experiments on the basis of previous reports (Ladner et al., 1988; Xu et al., 2000). Tetrazolium salt (MTS) solution as well as luciferase analysis reagents were bought from Promega (Sydney, NSW, Australia). TRAcP staining kit (Sydney, NSW, Australia) was purchased from Sigma Aldrich.

### Experimental Animals

Animal experiments of OVX were designed and approved by the Institutional Animal Care and Use Committees of Wenzhou Medical University (Ethic No. wydw2019–0247). The animals were fed in specific pathogen free laboratory facilities in a constant temperature environment and a 12 h light and 12 h dark condition.

### Bone Marrow Monocytes Culture

Bone marrow monocytes (BMMs) were obtained from the freshly isolated bone marrow of the femur and tibia of 8 week old C57BL/6J mice. The use BMMs for *in vitro* experiments was permitted by the Animal Ethics Committee, University of Western Australia (RA/3/100/1244). Then, BMMs were cultured in complete α-MEM (with additional 10% FBS, 100 U/ml penicillin, and 100 µg/ml streptomycin) in the presence of 50 ng/ml M-CSF with the change of medium every two to three days.

### *In vitro* Osteoclast Differentiation Assay

BMMs were cultured in 96-well plates overnight at a density of 5 × 10^3^ cells/well. Then the cells were activated in a complete α-MEM medium added with 50 ng/ml M-CSF and 50 ng/ml RANKL, supplemented without or with NOT (1, 2.5, 5 and 10 μM) every other day until osteoclasts took shape. After 5 days, the cells were immobilized with 4% paraformaldehyde (PFA) and then stained for TRAcP employing a leucocyte acid phosphatase staining kit based on manufacture’s instruction. TRAcP-positive cells consisting of more than three nuclei were regarded as osteoclasts and counted. What is more, the effects of NOT (10 μM) at different stages of osteoclasts differentiation were also evaluated. Cells were co-cultured with M-CSF and RANKL and then exposed to NOT at different time points from day 1 to day 5. At the end of the experiment, cells were fixed by 4% PFA and then stained for TRAcP.

### Cell Viability and Proliferation Assay

The MTS [3− (4, 5–dimethylthiazol–2–yl) -5–(3–carboxymethoxyphenyl)–2–(4-sulfophenyl)–2H–tetrazolium)] assay was conducted to detect the cell viability effect of NOT. BMMs were cultured at a concentration of 5 × 10^3^ cells/well in 96-well plate and stayed overnight for anchorage. The next day, the cells were dealt with different dose of NOT for 48 h. After that, the 96-well plate was incubated in 37°C incubator for 2 h with MTS solution (10 ul/well). Following that, the absorbance was analyzed by BMG plate reader (Ortenberg, Germany) at 490 nm wavelength.

### Hydroxyapatite Resorption Assay

For measuring the resorptive activity of osteoclasts, BMMs were cultured in a six well plate coated with collagen (BD Biosciences) at a density of 1 × 10^5^ cells/well and stimulated with 50 ng/ml M-CSF and RANKL for 3 days in order for osteoclasts development. Then, mature osteoclasts were seeded onto hydroxyapatite coated 96-well plate (Corning Osteoassay, Corning, NY, United States) at consistent cell numbers with completed α-MEM medium containing M-CSF as well as RANKL. 12 h after that NOT was added into the medium. Another 48 h later, half of cells were fixed with 4% PFA and stained for TRAcP activity for osteoclasts counting under a light microscope. The other half of cells were removed from resorbed areas with 10% bleach solution for 10 min. The images of resorbed areas were acquired by Nikon microscope (Nikon Corporation). ImageJ software was applied to quantify the percentage of resorbed area per osteoclast.

### Luciferase Reporter Assay

Luciferase reporter constructs (NFATc1, NF-κB, ARE) were used to transfect the RAW264.seven cells (American Type Culture Collection, Manassas, VA, United States), which, respectively, respond to NFATc1, NF-κB and ARE (Wang et al., 2003; van der Kraan et al., 2013; Liu et al., 2019). After transfection, RAW264.seven cells were cultured at a various density of 1.5 × 10^5^, 5 × 10^4^ and 1 × 10^5^ cells/well in 48-well plates overnight and pretreated with different doses of NOT for 1 h. Then the cells were stimulated with RANKL (50 ng/ml) for 24, 6 and 48 h (for measurement of NFATc1, NF-κB and ARE separately). Cells were lysed for luciferase activity measurement using the Promega Luciferase Assay System, in accordance with the guidance provided by manufacturer (Promega, Sydney, NSW, Australia).

### Immunofluorescence Staining and Confocal Microscopy

A number of 5 × 10^3^ BMMs each well were seeded in a 96-well plate in the presence of 50 ng/ml M-CSF overnight. The following day, BMMs were then treated with 50 ng/ml RANKL for 5 days to generate mature osteoclasts with or without different concentrations of NOT (5 μM or 10 μM). Cells were then fixed for 10 min using 4% PFA and blocked with 3% bovine serum albumin (BSA) for 30 min. Cells were then probed with rhodamine-conjugated phalloidin (Invitrogen, United States) for 45 min in the dark room to stain F-actin. For probing NFATc1 protein expression, BMMs were incubated with NFATc1 primary antibody for 2 h and then dyed with secondary antibody (Sigma-Aldrich, Australia) conjugated with Alexa Fluor-488 (Invitrogen, United States) for 30 min. After being rinsed with PBS and then stained with DAPI, cells were preceded for acquiring images by confocal fluorescence microscope (Nikon, A1 PLUS, Tokyo, Japan).

### Detection of Intracellular ROS Generation

6-carboxy-2’, 7’-dichlorodihydrofluorescein diacetate (carboxy-H2DCFDA) dye was used for detected intracellular ROS activity based on manufacture’s instruction (Molecular Probes, Australia). BMMs were cultured in a 96-well plate at a density of 1 × 10^4^ cells/well. After cells were adherent, the medium was changed with stimulating medium with 50 ng/ml RANKL and NOT at different concentrations (0, 5, and 10 µM). After 48 h, BMMs were starved for 1 h in Hanks balanced salt solution and then stained with Hanks balanced salt solution containing 20 μM carboxy-H2DCFDA in the dark for 45 min. The 2’, 7’-dichlorofluorescein (DCF) fluorescence was captured by confocal fluorescence microscope (Nikon, A1 PLUS, Tokyo, Japan). The numbers of ROS-positive cells per field were analyzed using ImageJ software.

### Quantitative Real-Time Polymerase Chain Reaction (qRT-PCR)

BMMs were added with different doses of NOT (0, 5 and 10 μM) for 5 days with M-CSF (50 ng/ml) and RANKL (50 ng/ml) to determine the expression of osteoclast-specific genes and ROS-related genes. Cells were lysed by Trizol reagent (Thermo Fisher Scientific) to extract total RNA. With an oligo-dT primer (Promega), 1 μg total RNA was reverse-transcribed into single-stranded cDNA. SYBR Green Master Mix (Imgenex, Littleton, CO, United States) was used to perform qRT-PCR in the present with the designed primers displayed as following: *TRAcP(Acp5) (*Forward:5’-CAGCAGCCAAGGAGGACTAC-3’; Reverse:5’-ACATAGCCCACACCGTTCTC-3’), *Calcitonin receptor* (Forward:5’-TGGTTGAGGTTGTGCCCA-3’; Reverse:5’-CTCGTGGGTT TGCCTCATC-3’), *V-ATPase-d2* (Forward: 5’-GTG​AGA​CCT​TGG​AAG​TCC​TGA​A-3’; Reverse:5’-GAGAAATGTGCTCAGGGGCT-3’), *Ctsk* (Forward: 5’-CCA​GTG​GGA​GCT​ATG​GAA​GA-3’; Reverse: 5’-AAG​TGG​TTC​ATG​GCC​AGT​TC-3’), *Hprt1*(Forward:5’-GTTGGGCTTACCTCACTGCT -3’; Reverse:5’-TAATCACGACGCTGGGACTG-3’), *Nrf2*(Forward:5’- TCT​CCT​CGC​TGG​AAA​AAG​AA-3’; Reverse: 5’-AAT​GTG​CTG​GCT​GTG​CTT​TA-3’), *Keap1*(Forward: 5’-TGC​CCC​TGT​GGT​CAA​AGT​G-3’; Reverse:5’- GGT​TCG​GTT​ACC​GTC​CTG​C-3’), *Gsr* (Forward: 5’-TGG​CAC​TTG​CGT​GAA​TGT​TG-3’; Reverse: 5’-TGT​TCA​GGC​GGC​TCA​CAT​AG-3’), *NQ O 1*(Forward:5’- TTC​TCT​GGC​CGA​TTC​AGA​G-3’; Reverse:5’ -GGC​TGC​TTG​GAG​CAA​AAT​GAG-3’), *Catalase* (Forward:5’-CTCGCAGAGACCTGATGTCC-3’; Reverse:5’- GAC​CCC​GCG​GTC​ATG​ATA​TT-3’). Relative mRNA levels were normalized to expression of the housekeeping gene *Hprt1*. The results of mentioned gene were obtained from the ViiA™ 7 PCR machine (Applied Biosystems, United Kingdom).

### Western Blotting Assay

For analysis of long-term effects on osteoclast-related proteins, BMMs were isolated and cultured at a density of 8 × 10^4^ cells/well in complete medium with M-CSF in 6-well plates with or without NOT (10 μM) in the presence of RANKL (50 ng/ml) for 0, 1, 3, 5 days. In order to activate osteoclast-specific signaling pathways, BMMs were pretreated in the absence or presence of NOT (10 μM) for 1 h before RANKL stimulation for 0, 5, 10, 20, 30 and 60 min. Cells were lyzed completely using radioimmune precipitation (RIPA) buffer to obtain protein extraction. Proteins were then subjected to 10% sodium dodecyl sulfate-polyacrylamide gel electrophoresis (SDS-PAGE) and transferred to a nitrocellulose membrane (GE Healthcare, Silverwater, NSW, Australia). The membranes were blocked in 5% skim milk in Tris-Buffered Saline and Tween 20 (TBST) buffer containing 0.1% Tween-20 (Sigma, United States) for 1 h, and incubated overnight at 4°C with various primary antibodies (1:1000) as following: p-P38, p-JNK1/2, P38, JNK, IκB-ɑ, NFATc1, MMP9, c-Fos, CTSK, HO-1, Catalase and β-actin. Consequently, the membranes were washed and incubated for 1 h with the corresponding horseradish peroxidase-conjugated secondary antibodies. Finally, the protein bands were visualized using Enhanced Chemiluminescence reagents (Perkin Elmer, Waltham, MA, United States) and analyzed by ImageJ software.

### Measurement of Intracellular Ca^2+^ Oscillation

BMMs (1×10^4^ cells/well) were cultured in a 48-well plate with or without treatments according to different groups. In the negative control group, cells were incubated only with M-CSF (50 ng/ml). In the positive control group, cells were incubated with 50 ng/ml M-CSF and RANKL in the absence of NOT. While in the treatment group, cells were dealt with 50 ng/ml M-CSF and RANKL as well as indicated concentration of NOT (10 μM). After 24 h, the cells were rinsed three times with assay buffer (HANKS balanced salt solution added with 1 mM probenecid and 1% FBS), and incubated with Fluo-4 staining solution (Fluo4-AM dissolved in 20% (w/v) pluronic-F127in DMSO diluted in assay buffer) in the dark room at 37°C for 45 min. When staining was completed, the cells were washed once in assay buffer. The fluorescence intensity was observed with fluorescent light (at an excitation wavelength of 488 nm) by inverted fluorescence microscope (Nikon). Images were acquired every 2 s for 3 min. Cells with at least two oscillations were regarded as oscillating cells. The average amplitude of individual oscillating cell was analyzed by Nikon Basic Research Software (Nikon).

### Ovariectomized Mice Model

Fifteen female C57BL/6 mice (8 weeks-old; 18.18 ± 0.07 g) offered by the Animal Center of the Chinese Academy of Science (Shanghai, China) were randomly assigned into three equal groups: sham operated mice, OVX mice, NOT (10 mg/kg) treated OVX mice. One week after adaptive feeding, the OVX mice and NOT treated OVX mice were treated with 10% chloral hydrate solution and bilateral ovaries were removed, while the ovaries were only raised in the sham operated mice. One week after surgery, PBS or NOT (10 mg/kg) was injected intraperitoneally every two days for 6 weeks. All treated mice were euthanized 1 day after last administration. The femurs were removed, fixed in 10% formalin for 24 h and radiologically analyzed by micro-computed tomography (micro-CT) and bone histomorphometric analysis.

### Micro-CT Analysis

The femur samples were fixed and then scanned by Skyscan 1176 micro-CT equipment (Skyscan, Aartselaar, Belgium). Images were obtained using a 50 kV X-ray tube voltage, a 500 μA current, an isotropic pixel size of 9 μm and a 0.5 mm-thick aluminium filter for beam hardening. NRecon Reconstruction software (Bruker micro-CT) was employed to reconstruct images. A refined volume of 0.5 mm below the growth plate and 1 mm in height was then selected for further analysis using DATAVIEWER software program (Bruker micro-CT). Parameters, including the bone volume/tissue volume (BV/TV), trabecular thickness (Tb.Th), trabecular number (Tb.N) and trabecular spacing (Tb.Sp), were calculated by CTAn software (Bruker micro-CT, Kontich, Belgium). While cortical bone area (Ct. Ar), total tissue area (Tt. Ar), cortical area fraction (Ct. Ar/Tt. Ar) and cortical thickness (Ct. Th) were applied to observe the cortical bone. As for cortical bone analysis, a volume of interest (5 mm above the growth plate of distal femur and 1 mm in height) was chosen.

### Bone Histomorphometric Analysis

The femur samples were fixed in 10% formalin, then rinsed three times using 1×PBS. After that, femurs were decalcified with 14% ethylenediaminetetraacetic (EDTA) for 14 days. Samples were then embedded in paraffin blocks which were cut into sequential 5 μm-thick sections to perform Hematoxylin and eosin (H and E) and TRAcP staining. Bone histomorphometric analysis was performed by quantifying parameters including BV/TV and osteoclast surface/bone surface (Oc. S/BS) using BIOQUANT OSTEO software (Bioquant Image Analysis Corporation, Nashville, TN, United States) in the basis of a method proposed by Sawyer et al. (Sawyer et al., 2003).^26^

### Statistical Analysis

All data presented are obtained from at least three independent experiments. Data are shown as means ± standard error of mean (SEM). Statistical significance between results was determined by Student’s t test and ANOVA. Student’s *t* test was conducted to test the significance of difference between two groups, while One-way ANOVA used for more than two groups. In addition, different treatment groups were evaluated by two-way ANOVA. *P < 0.05* was regarded statistically significant.

## Results

### Notopterol Attenuates Osteoclast Differentiation *in vitro*

To exclude the cytotoxic effect of NOT, MTS assay was used to assess cell viability. We found that NOT does not affect the viability of BMMs (Figure 1B) and mature osteoclast (Supplementary Figure S1). In order to investigate the effect of NOT on RANKL-induced osteoclastogenesis, freshly isolated BMMs were incubated with different concentrations of NOT under the stimulation of RANKL. The result indicated that osteoclast formation declined with the increment of NOT concentrations. The total number and size of multinucleated osteoclast was conspicuously reduced with NOT concentrations increased from 5 to 10 μM (Figures 1C,D). Osteoclast nuclear fusion is believed to be an essential step in the generation of multinucleated osteoclasts. In order to detect the influence of NOT on morphological changes in actin cytoskeletal structure, rhodamine-phalloidin was applied to observe the structures of F-actin belt and DAPI staining was used to identify the sum of nuclei per osteoclast. Mature osteoclasts are presented with the occurrence of clearly defined F-actin belt with multiple nuclei after inducing by RANKL. It was apparently displayed by the results that NOT untreated cells had well-defined F-actin belts with numerous nuclei formed in mature osteoclasts. On the contrary, NOT exposed cells possessed fewer nuclei as well as smaller size with F-actin belt disruption (Figure 1E). Consistently, the number of nuclei each osteoclast and the mean area of per osteoclast were also remarkably diminished after NOT treatment (Figures 1F,G), suggesting that NOT might interfere with precursor cell fusion during osteoclast formation. Additionally, we found that NOT was able to inhibit the osteoclastogenesis on a time-dependent manner (Supplementary Figures S2A–C), especially in the early and middle stage. Besides, alkaline phosphatase (ALP) staining assay was performed to probe the role of NOT in osteogenesis. As shown in Supplementary Figures S3A,B, bone formation mediated by osteoblasts was rarely affected by NOT even at the concentration of 10 μM. Taken together, these results revealed that NOT has a predominantly suppressive impact on RANKL-induced osteoclastogenesis as well as osteoclast fusion in both dose and time dependent ways. Meanwhile, NOT has no influence on the viability and proliferation of BMMs as well as the osteoblast differentiation of MC3T3-E1 cells.

### Notopterol Suppresses Osteoclast Hydroxyapatite Resorption

**FIGURE 2:**
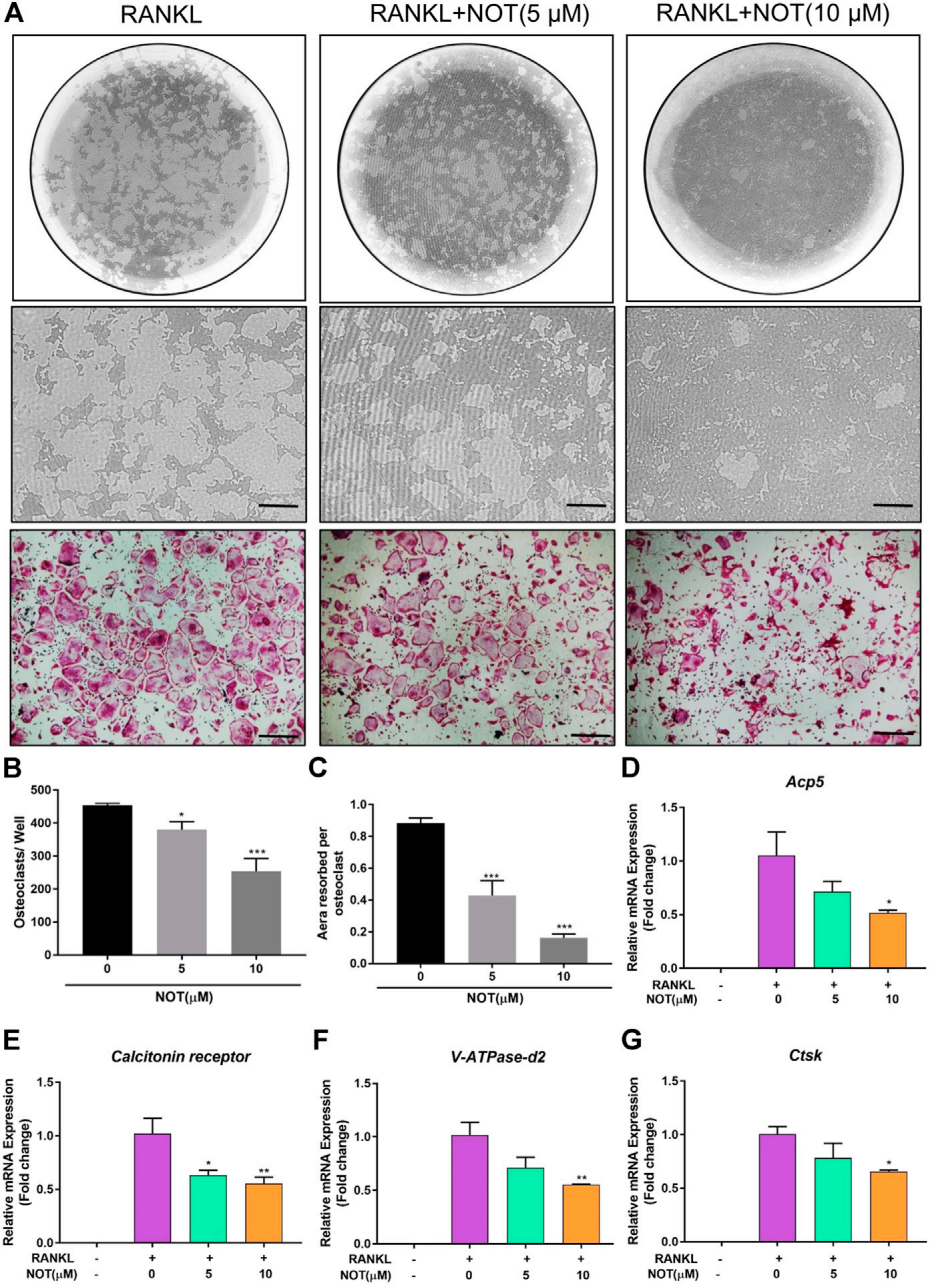
Notopterol suppresses osteoclast hydroxyapatite resorption and expression of osteoclast marker genes. **(A)** Representative images of the hydroxyapatite surface after removal of osteoclasts with corresponding TRAcP-stained osteoclasts. Scale bar = 200 μm. Representative images showing the effect of 10 μM Notopterol treatment on indicated days. **(B)** Quantification of TRAcP-positive multinucleated cells (nuclei >3). **(C)** Quantification of the percentage area of hydroxyapatite surface resorbed per osteoclast. qRT-PCR analysis was performed to detect osteoclast-specific genes *Acp5* **(D)**, *Calcitonin receptor* **(E)**, *V-ATPase-d2* **(F)** and *Ctsk* **(G)**. The expression levels of these genes were normalized to the expression of *Hprt1*. “−” means RANKL untreated; “+” means RANKL treated; Data are presented as mean ± SEM. All *in vitro* experiments were repeated three times with similar results. *p < 0.05, **p < 0.01, ***p < 0.001 relative to RANKL-induced controls.

To explore the impact of NOT on osteoclast resorptive activity, equivalent numbers of osteoclasts were cultured onto the hydroxyapatite-coated plate. After exposing mature osteoclasts with different concentrations of NOT (0, 5 and 10 μM) for 48 h, the percentage of resorption areas was determined. As depicted in Figures 2A,B, the number of mature osteoclasts was distinctly decreased by 5 and 10 μM NOT treatment, which is likely due to the effect of NOT on osteoclast formation. Moreover, a dose-dependent repressive effect on hydroxyapatite resorption activity was observed in contrast to the RANKL group (Figure 2C). These results indicated that NOT acts as an inhibitor to osteoclast resorptive activity.

### Notopterol Down-Regulates the Expression of Osteoclast-Relevant Genes

Next, we explored the impact of NOT on RANKL-induced genes expression during osteoclastogenesis. The mRNA expression of osteoclast-relevant genes including *Acp5*, *Calcitonin receptor*, *V-ATPase-d2* and *Ctsk*, which involved in osteoclast differentiation, fusion and resorption, were dramatically down-regulated during RANKL-induced osteoclastogenesis following exposure to NOT dose-dependently (Figures 2D–G). Collectively, these results were in line with the restraining effects of NOT on osteoclast differentiation as well as resorption activity.

### Notopterol Abrogates RANKL-Mediated NFATc1 Activation and Associated Downstream Protein Expression

**FIGURE 3:**
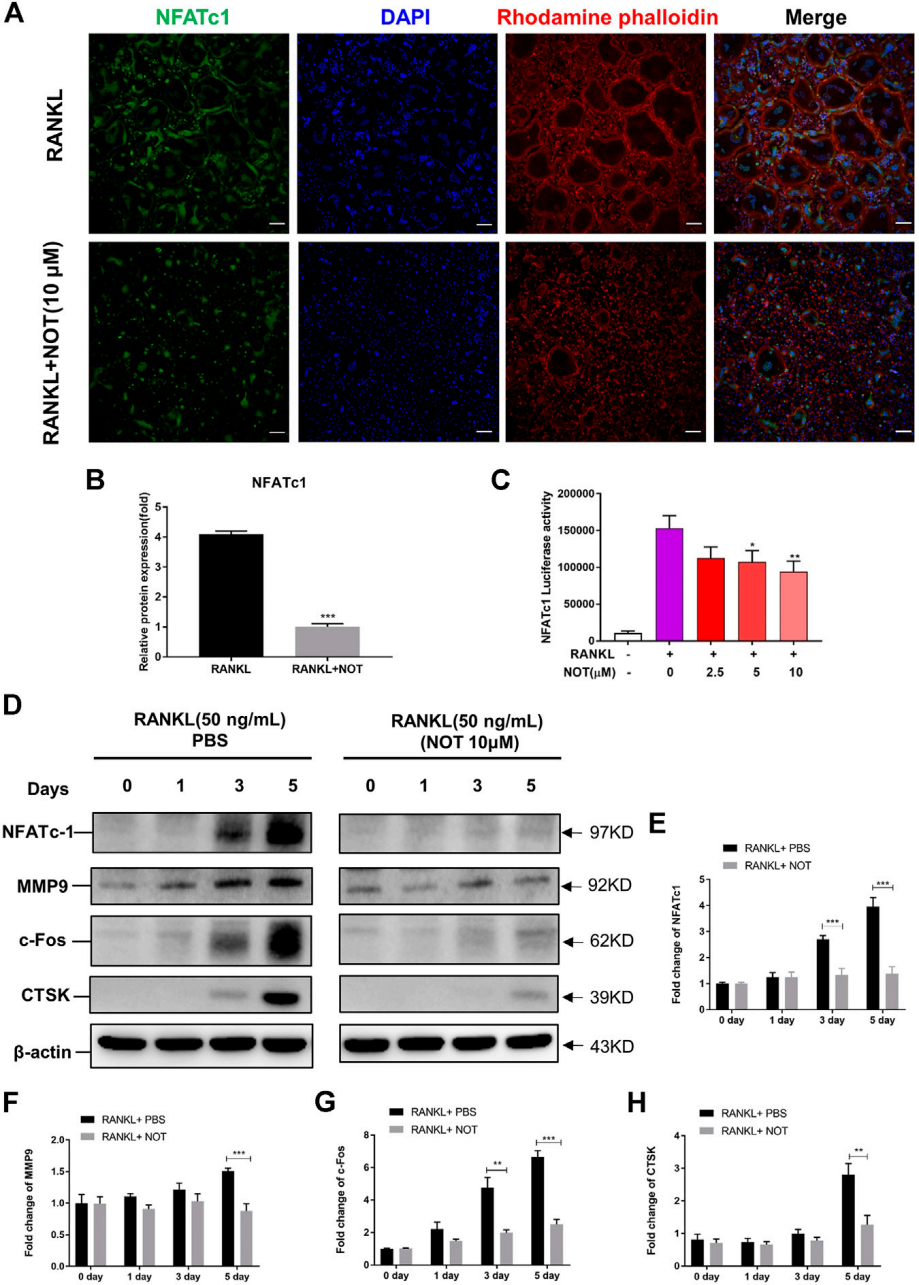
Notopterol abrogates RANKL-induced NFATc1 activation and associated downstream protein expression. **(A,B)** Representative confocal images **(A)** and quantification analysis **(B)** indicated that NFATc1 protein expression activated by RANKL was strikingly hampered by Notopterol. Scale bar = 200 μm. **(C)** RAW 264.7 cells transfected with an NFATc1 luciferase construct were pre-treated with indicated concentrations of Notopterol, followed by RANKL (50 ng/ml) stimulation for 24 h. NFATc1 luciferase activity was measured with a luciferase reporter assay system. **(D)** Representative images of Western blotting reflecting the effects of Notopterol on NFATc1, MMP9, c-Fos, CTSK induced by RANKL. **(E–H)** The ratios of band intensity of NFATc1 **(E)**, MMP9 **(F)**, c-Fos **(G)**, CTSK **(H)** relative to β-actin were quantitatively determined. “−” means RANKL untreated; “+” means RANKL treated. Data are presented as mean ± SEM. All *in vitro* experiments were repeated three times with similar results. Significant differences between the control and treatment groups are shown as *p < 0.05, **p < 0.01 and ***p < 0.001.

To further determine the role of NOT on RANKL-mediated pathways, we firstly analyzed its impact on RANKL-induced activation of NFATc1. As shown in the immunofluorescence staining, NFATc1 protein expression was strikingly hampered by NOT at day five (Figures 3A,B). In addition, NOT hampered RANKL-induced NFATc1 activation dose-dependently by measuring luciferase activity, apparently in the concentration of 10 μM (Figure 3C). Subsequently, we examined whether NOT exerts an inhibitory role on NFATc1 protein expression. As expected, NFATc1 itself and related downstream protein expression such as c-Fos, matrix metallopeptidase 9 (MMP9) and CTSK were completely blocked by NOT (10 μM) treatment on Day five (Figures 3D–H).

### Notopterol Attenuates RANKL-Mediated NF-κB, MAPK and Calcium Signaling Pathways

**FIGURE 4:**
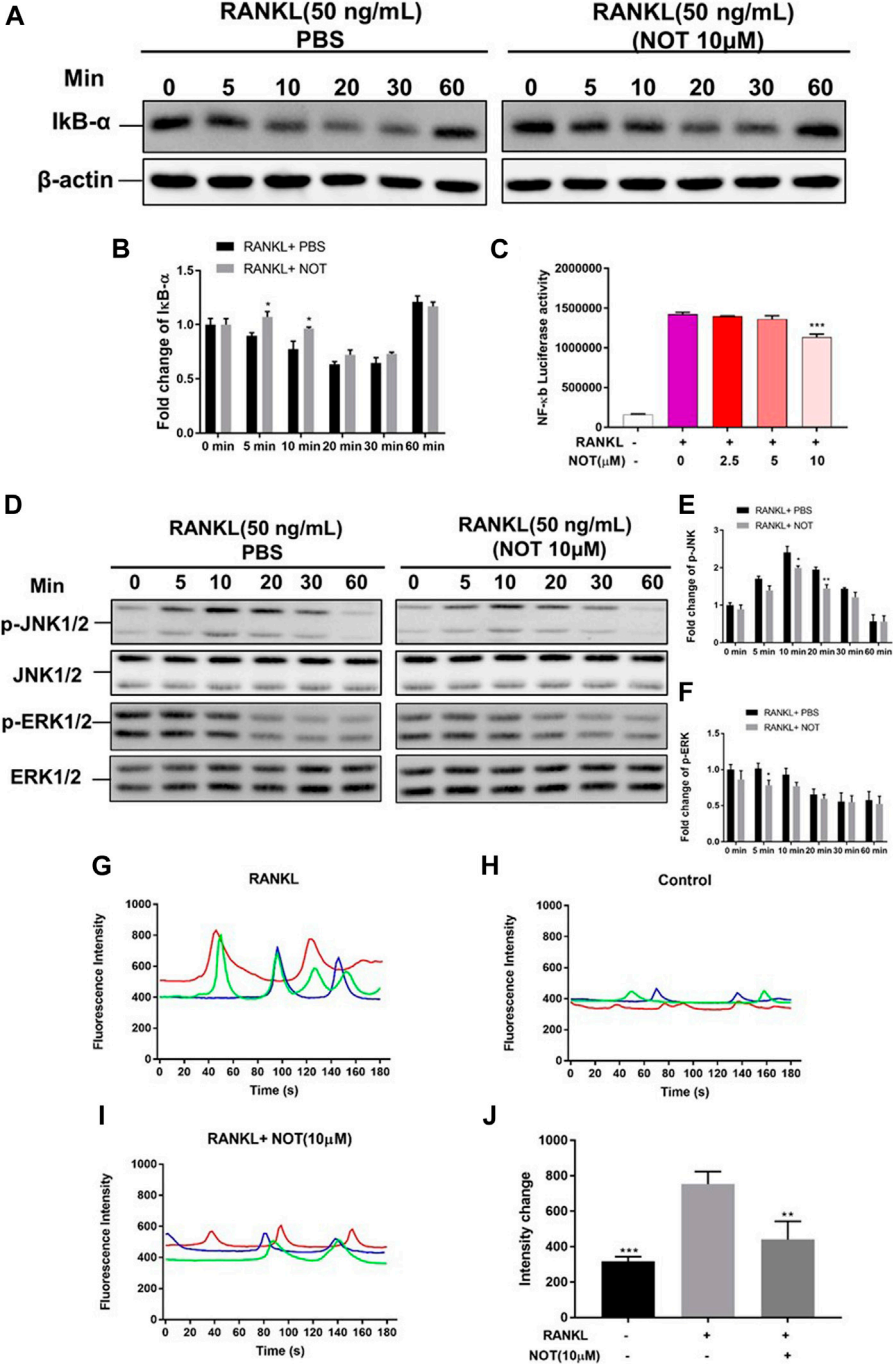
Notopterol represses NF-κB, MAPK activation and calcium oscillation during osteoclastogenesis. **(A,B)** Representative Western blotting images **(A)** and quantification analysis **(B)** of IκB-α from BMMs which were induced with M-CSF and RANKL in the presence of NOT (10 μM). **(C)** Luciferase activity in RANKL stimulated RAW264.7 cells transfected with an NF-κB luciferase construct. NF-κB luciferase activation was decreased following exposure to various concentration of Notopterol. **(D)** Representative images of western blots suggesting the expression level of p-JNK1/2 and p-ERK1/2 normalized to JNK1/2 and ERK1/2. **(E,F)** Quantitative analysis of the fold change in p-JNK1/2 and p-ERK1/2 expression after Notopterol treatment. **(G–J)** Representative images of Ca^2+^ oscillation pattern stimulated by RANKL **(G)**, negative control (M-CSF only) **(H)**, 10 μM Notopterol treatment prior to RANKL stimulation **(I)**. **(J)** Quantification of intensity of Ca^2+^ oscillation captured across multiple cells for each condition and maximum peak intensity minus baseline intensity (*n* = 3). Data are presented as mean ± SEM. All *in vitro* experiments were repeated three times with similar results. Significant differences between the control and treatment groups are shown as *p < 0.05, **p < 0.01 and ***p < 0.001.

Evidence indicated that the activation of NFATc1 relies on RANKL-induced NF-κB as well as MAPK pathways. IκB-α, an inhibitor of NF-κB, was also involved to investigate the upstream signaling pathway before NF-κB activation. Western blotting depicted that NOT has an inhibitory effect on degradation of IκB-α at 5 and 10 min in comparison to the RANKL-only group (Figures 4A,B), whereas p-P65 was not apparently affected (Supplementary Figures S4A, B). Additionally, luciferase reporter assays were conducted to probe the impact of NOT on RANKL-induced NF-κB activity. NF-κB luciferase activation was decreased following exposure to NOT at the concentration of 10 μM (Figure 4C). Finally, we identified that the expression of p-JNK1/2 was markedly repressed at 10 and 20 min and the expression of p-ERK1/2 was distinctly inhibited at 5 min after 10 μM NOT treatment (Figures 4D–F), whereas p-P38 was not significantly affected (Supplementary Figures S5A, B).

RANKL stimulation gives rise to the activation of Ca^2+^ signal transduction pathways, which triggers Ca^2+^ oscillations that lead to NFATc1 self-amplification and nuclear translocation. Given that NOT is able to attenuate NFATc1 activity, we further determined the impact of NOT on Ca^2+^ oscillations in the cytoplasm. As anticipated, treatment with RANKL induced Ca^2+^ oscillations whereas extremely weak Ca^2+^ flux was seen in the group of absent RANKL (Figures 4G,H). Noticeably, NOT distinctly decreased the amplitude of Ca^2+^ oscillations mediated by RANKL (Figures 4I,J). The expression of phosphorylated phospholipase C (PLC) gamma2 was detected by western blotting at least three times. Unfortunately, we have not obtained the satisfactory results (Supplementary Figure S6). These results suggested that NOT constrains the Ca^2+^ signaling pathway mediated by RANKL. To sum up, these data demonstrated that NOT blocks RANKL-induced NF-κB, MAPK and calcium signaling pathways.

### NOT Depresses RANKL-Induced ROS Generation and Enhances ROS Scavenging Enzymes Expression

**FIGURE 5:**
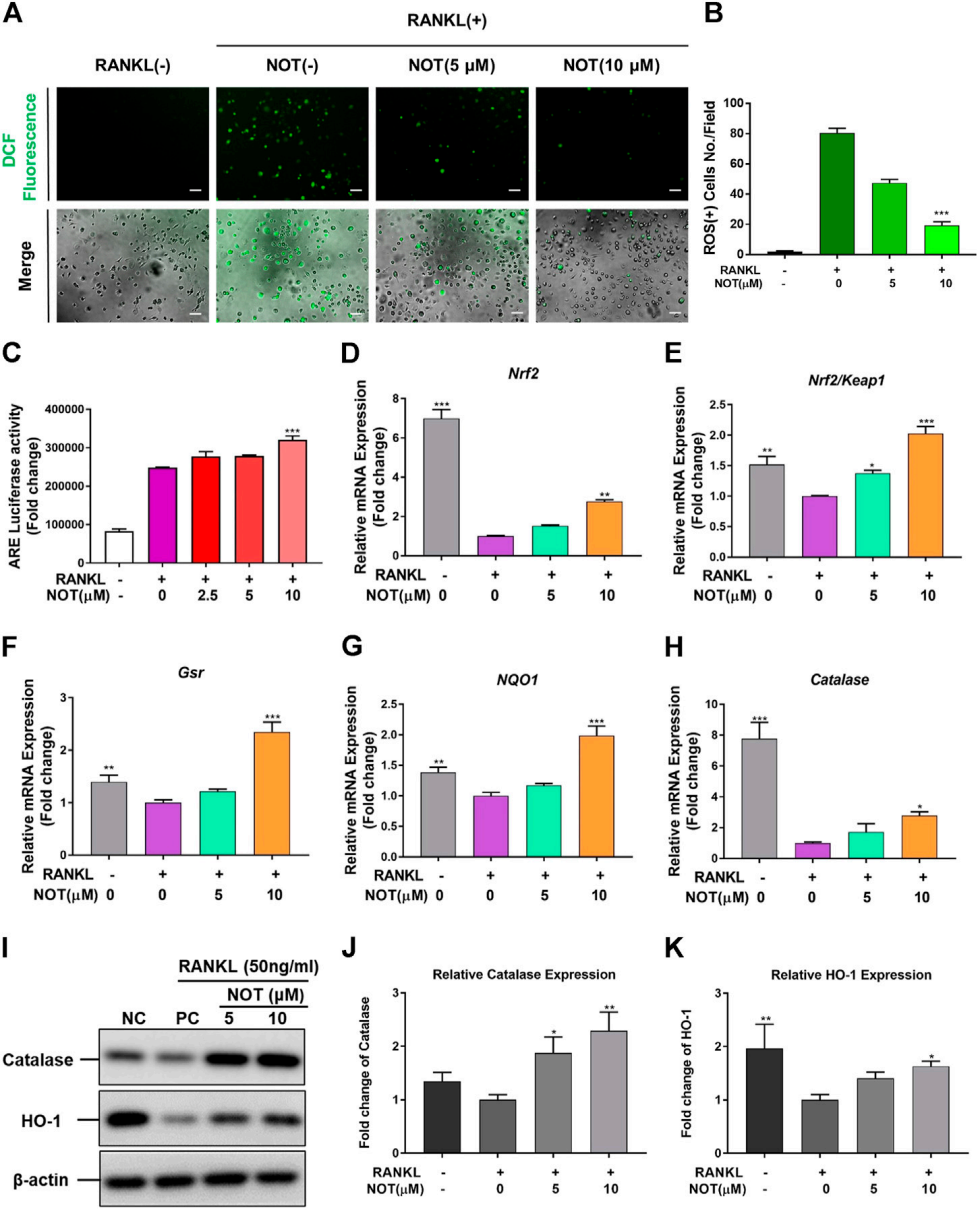
Notopterol suppresses RANKL-induced ROS generation and enhances ROS scavenging enzymes expression. **(A)** Representative confocal images of RANKL-induced ROS generation in cells with or without the addition of NOT. **(B)** Quantification of the average number of ROS-positive cells per field (*n* = 3). **(C)** Antioxidant response element luciferase activity was lifted with the higher Notopterol concentration indicated by analyzing luciferase assay. **(D–H)** qRT-PCR analysis was conducted to determine the expression of ROS scavenging enzymes including Nrf2 **(D)**, Nrf2/Keap1 **(E)**, Gsr **(F)**, NQO1 **(G)** and catalase **(H)**. The expression levels of these genes were normalized to the expression of Hprt1. **(I–K)** The protein expression of catalase and HO-1 after treatment by Notopterol (5 and 10 μM) with or without RANKL (50 ng/ml). The statistical significance of differences in protein expression among the four groups was analyzed. The expression of the above proteins was determined relative to β-actin expression. Data are presented as mean ± SEM. All *in vitro* experiments were repeated three times with similar results. Significant differences between the control and treatment groups are shown as *p < 0.05, **p < 0.01 and ***p < 0.001.

Accumulating evidence has shown that ROS plays a crucial part in the process of osteoclasts formation under the stimulation of RNAKL (Agidigbi and Kim., 2019). Therefore, we next explored whether NOT could affect ROS production in BMMs. Intracellular ROS generation was probed by a carboxy-H2DCFDA dye and the DCF fluorescence was captured using confocal microscopy. We found that the intensity of DCF fluorescence per positive cell was obviously declined in a dose-dependent manner in the NOT treatment group compared with the RANKL group (Figures 5A,B). Nrf2/Keap1/ARE pathway is a key mechanism in regulating the level of ROS by governing an array of cytoprotective genes (Yagishita et al., 2014). As a *cis*-regulatory element, ARE exists in the promoter area of some genes encoding cytoprotective proteins. Under the stimulation of RNAKL, the activity of ARE was gradually lifted with the higher NOT concentration indicated by luciferase assay (Figure 5A). Nrf2 is an essential transcription factor, and a primarily regulatory factor of oxidative stress, while Keap1 interacts with Nrf2 and mediates Nrf2 function. The Nrf2/Keap1 pathway chiefly regulates the antioxidant enzymes, including catalase, NAD(*P*)H quinine oxidoreductase 1 (NQO1), hemeoxygenase-1 (HO-1) and glutathione reductase (Gsr) (Taguchi et al., 2011; Kanzaki et al., 2013; Wang et al., 2017). By analyzing qRT-PCR and western blotting, it was noticed that the gene and protein expression of antioxidant enzymes were down regulated by RANKL but elevated under the treatment of NOT (Figures 5C–K). These data demonstrated that NOT reduces RANKL-induce ROS production through enhancing antioxidant enzymes expression.

### Notopterol Prevents OVX Induced Trabecular Bone Loss in Mice

**FIGURE 6:**
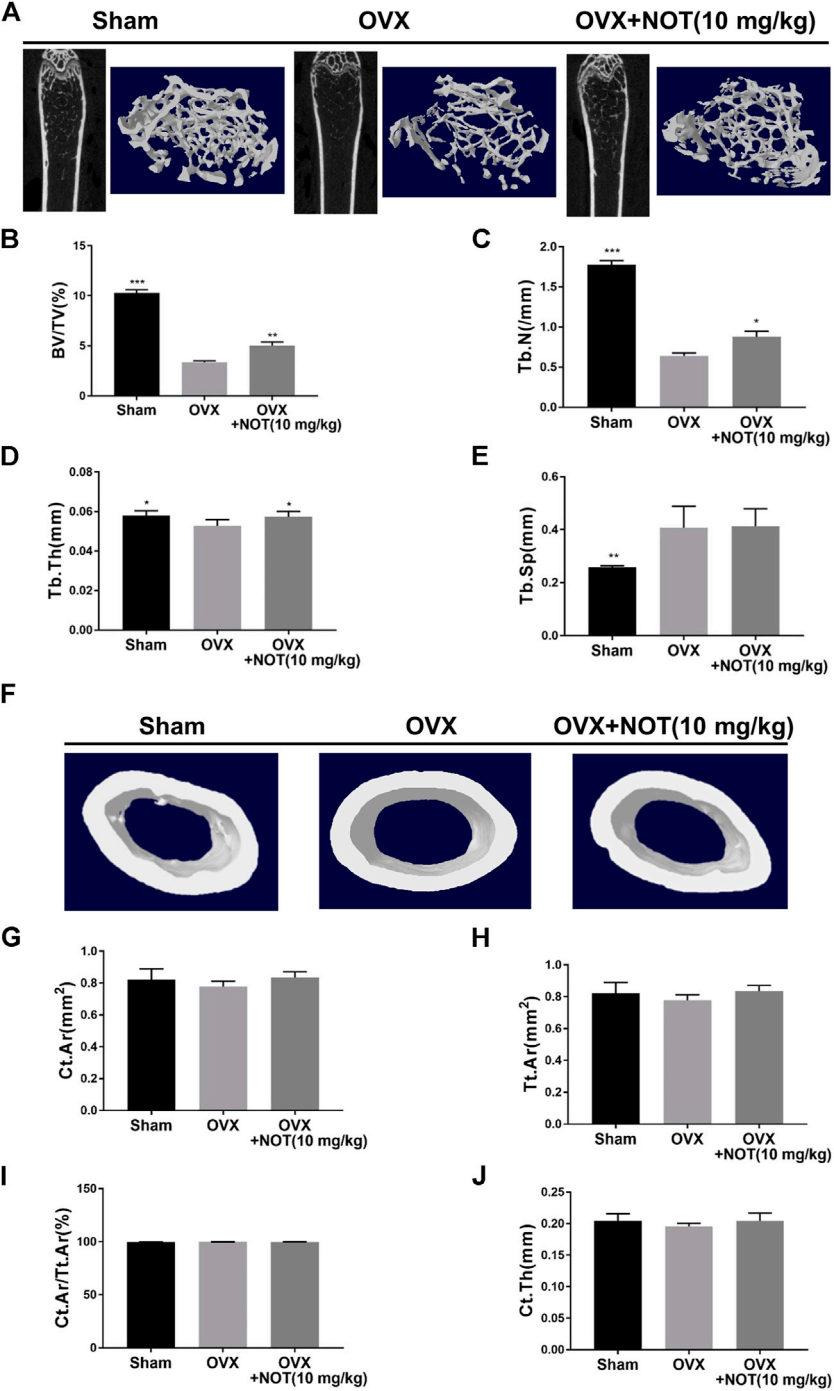
Notopterol treatment prevents bone loss in OVX-induced mice *in vivo*. **(A)** Representative 2-dimensional and 3-dimensional reconstruction micro-CT images of trabecular bone microarchitecture in the different groups. Trabecular bone was analyzed by micro-CT in sham-operated, ovariectomized mice after vehicle or Notopterol treatment (10 μM). **(B–E)** Trabecular bone volume/tissue volume fraction **(B)**, trabecular number **(C)**, trabecular thickness **(D)** were dramatically up-regulated after exposure to Notopterol. Trabecular separation has no obvious difference among these groups **(E)**. **(F)** Representative 3-dimensional reconstructed region of cortical bone suggested no difference among three groups in the femur. Cortical bone area **(G)**, total tissue area **(H)**, cortical area fraction **(I)** as well as cortical thickness **(J)** were also less different in the absent or present Notopterol groups. Data are presented as mean ± SEM. *n* = 5 per group. *p < 0.05, **p < 0.01 and ***p < 0.001 relative to the OVX group.

With the promising results *in vitro*, we further employed the OVX mouse model to investigate the impact of NOT on pathological bone loss *in vivo*. Micro-CT analysis revealed that OVX induces a significant trabecular bone loss, whereas treatment with NOT protects against trabecular bone loss in OVX group (Figure 6A). Quantitative analysis affirmed that bone parameters including BV/TV, Tb. N and Tb. Th were strikingly elevated in the NOT treated group in comparison to the OVX group (Figures 6B–D). However, the value of Tb. Sp has no obvious difference between OVX-induced mice group and NOT treatment group (Figure 6E). In contrast, reconstructed region of cortical bone seemed no difference among three groups (Figure 6F). Similarly, quantitative analysis also indicated that Ct. Ar, Tt. Ar, Ct. Ar/Tt. Ar as well as Ct. Th were insignificantly different in the absent or present NOT groups (Figures 6G–J).

**FIGURE 7:**
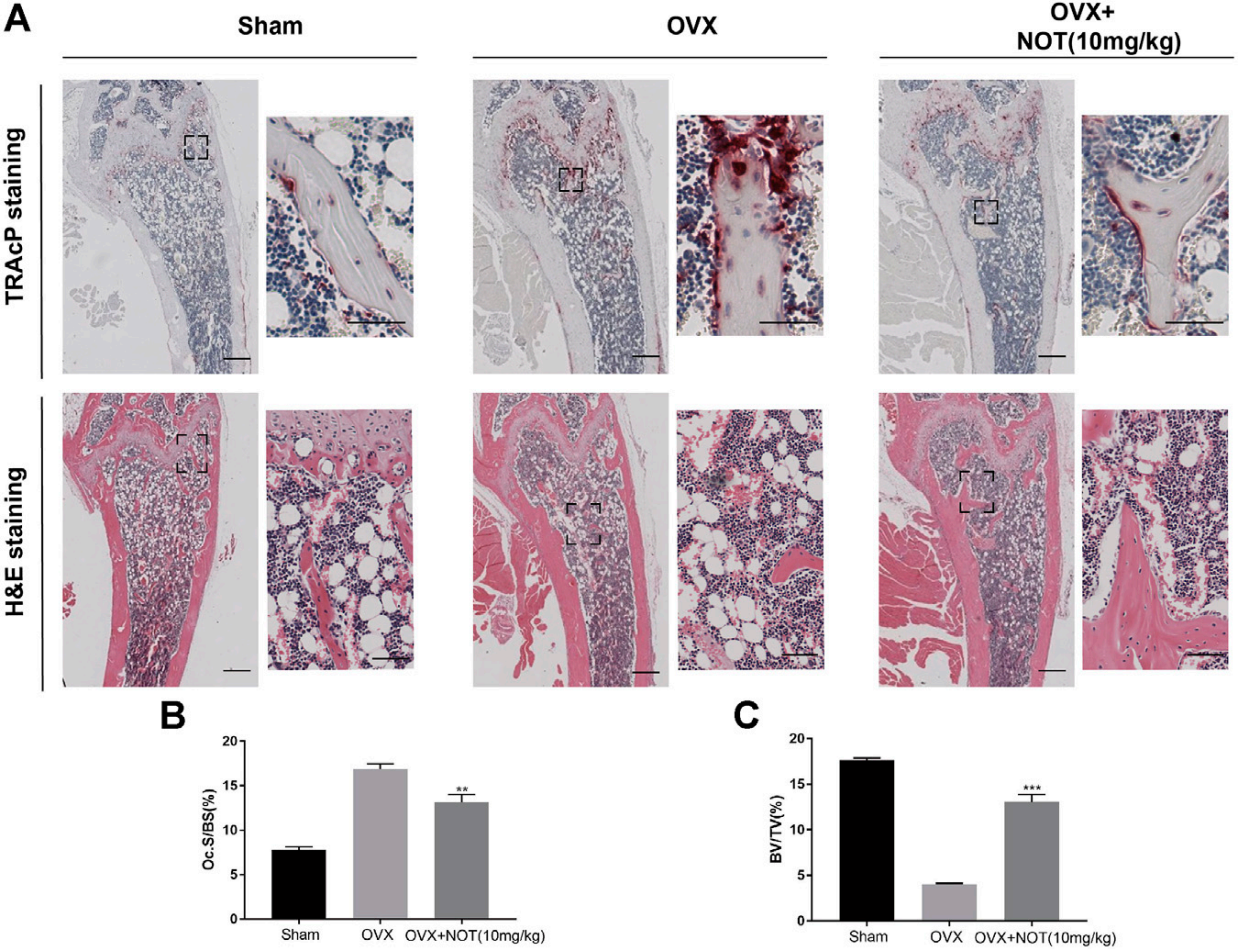
Notopterol ameliorates OVX-induced bone loss by suppressing osteoclast activity. **(A)** Representative images of decalcified bone stained with TRAcP and H and E in sham-treated, OVX, OVX + Notopterol (10 mg/kg) groups. Scale bar = 500 μm; Scale bar = 100 μm (TRAcP) and Scale bar = 200 μm (H and E) in the enlarge pictures. **(B–C)** Quantitative analysis of osteoclast surface/bone surface **(B)** and bone volume/total volume **(C)** in tissue sections. Data are presented as mean ± SEM. *n* = 3 per group. Significant differences between the control and treatment groups are shown as **p < 0.01 and ***p < 0.001.

Histological staining using H and E and TRAcP were performed to confirm the bone volume, bone surface area and osteoclasts surface area *in vivo*. The results revealed that quantification of osteoclast parameter, osteoclasts surface area per bone surface area, was enhanced in OVX group compared to the control group, but NOT considerably decreased the value of Oc. S/BS (Figures 7A,B). The value of BV/TV in the OVX + NOT mice was conspicuously higher than that of the OVX mice (Figure 7C). In summary, our data suggested NOT plays an active role in preventing OVX-mediated bone resorption by suppressing osteoclastogenesis *in vivo*.

## Discussion

Osteoporosis has become a crucial public health problem all over the world. Study has indicated that there are 5.5 million men and 22 million women diagnosing with osteoporosis. The economic burden of fragility fractures is tremendous, and costs of disease management are expected to increase by 25% in 2025 in European countries (Hernlund et al., 2013). The treatment and prevention of osteoporosis has become an urgent problem in the medical community. For the treatment of perimenopausal osteoporosis in women, hormone replacement therapy (HRT) has a significant effect on the treatment and prevention of osteoporosis. However, as early as 2001 from Woman’s Health Initiative survey, important warnings were raised that the HRT may increase the threat of cardiovascular disease and breast cancer (Cuzick, 2001). Bisphosphonates and denosumab have been shown to give rise to osteonecrosis of the jaw (Hamadeh et al., 2015; Khan et al., 2015; Boquete-Castro et al., 2016), and teriparatide can increase the risk of osteosarcoma (Gilsenan et al., 2018). Compared with synthetic chemicals, natural compounds seem to have an advantage in terms of safety, some of which inhibit bone resorption and bone loss in OVX mice (Jin et al., 2019; Zhou et al., 2019; Zhou et al., 2020). In this study, we explored the therapeutic effects of NOT on osteoclasts and OVX-mediated bone loss.

The formation and differentiation of mature osteoclasts from bone marrow macrophages requires the critical cytokines M-CSF and RANKL that are secreted by osteoblasts and stromal cells (Udagawa et al., 1999). The effect of M-CSF on osteoclastogenesis involves the survival, proliferation and differentiation of early progenitors into mature phagocytes. The effect of RANKL on osteoclastogenesis was confirmed by a research which reported that mice lacking the RANKL encoding gene displayed an osteopetrotic phenotype, on account of the deficiency of mature osteoclasts (Kong et al., 1999). As an important cytokine of osteoclastogenesis and osteoclast function, RANKL has become a new target for the therapy of osteoporosis and other osteolytic diseases. Our results revealed that NOT strikingly suppressed RANKL-mediated osteoclast formation and differentiation in a dose-dependent fashion without affecting the viability of BMMs. The hydroxyapatite resorption area was distinctly decreased after NOT treatment, suggesting that the impact of NOT was both on osteoclastogenesis and bone resorption activity.

NF-κB is one of the most crucial pathways to promote osteoclast formation and functions following the binding of RANKL to its receptor RANK. In the canonical NF-κB signaling pathway, IκB-α is phosphorylated by the activated Iκb kinase complex, resulting in ubiquitination leading to subsequent degradation of IκB-α and activation of mature subunit NF-κB1 (P50) and RelA (P65). This results in NF-κB complex (P50 and P65) translocating to nucleus to bind to its target genes (Hayden and Ghosh., 2004). As shown in our Western blot analysis as well as luciferase reporter gene assay, both the degradation of IκB-α as well as activation of NF-κB were suppressed by NOT without making a difference to phosphorylation of P65. Moreover, MAPKs, another well-known signaling pathway of TRAF6 recruitment during osteoclastogenesis, is a large family signaling molecules which consist of JNKs, ERKs and P38 (Chang and Karin., 2001; Wada and Penninger., 2004). Some studies have shown that they are essential for the survival of osteoclasts (Davis, 2000; Lee et al., 2001; Thouverey and Caverzasio., 2015). As expected, the phosphorylation of JNK and ERK were greatly stimulated by RANKL, whereas NOT attenuated the phosphorylation of JNK and ERK signaling pathways without affecting P38.

The NFATc1 transcription factor was originally discovered in activated T cells (Shaw et al., 1988) and had been demonstrated to be a vital downstream osteoclastogenic transcription factor which is highly induced by RANKL. The results of our study displayed that the protein expression level and NFATc1 transcription factor activation by RANKL were reduced by NOT. Furthermore, calcium signaling pathway is another primary pathway which is essential for the induction of NFATc1. It is evident that intracellular calcium oscillation contributes to the auto-amplification of NFATc1 (Takayanagi et al., 2002). We found that NOT only inhibited the intensity of calcium oscillation that stimulated in the presence of RANKL, but also blocked the expression of NFATc1. After down-regulating NFATc1 expression, various genes involved in osteoclast differentiation, fusion, and activation, including Acp5, calcitonin receptor, V-ATPase-d2 and Ctsk were also decreased with NOT concomitantly. PLC gamma2 phosphorylation was indicated to be related to cytosolic calcium increase (Gemma et al., 2018). However, we have not obtained the satisfactory result in p- PLC gamma2, which may be a limitation of this study.

Recently, increasing studies have revealed that ROS are involved in the process of osteoclastogenesis (Callaway and Jiang., 2015; Huang et al., 2015; Chen et al., 2019). ROS are able to affect the activity of NF-κB by down-regulating the phosphorylation of IκB-α (Hyunil et al., 2004). In addition, the expression of NFATc1 is also related to the activity of ROS. The production of ROS induced by RANKL can induce calcium oscillation, thus starting the activation of NFATc1 and its own amplification process (Min et al., 2010). In our study, NOT is capable of significantly inhibiting the production of ROS in the cytoplasm to affect the differentiation of osteoclasts. Nrf2, a transcriptional activator combined with anti-oxidant elements, can enhance the expression of antioxidant enzymes, such as NQO-1, HO-1 (Holmstrom et al., 2016; Zhang et al., 2017). In addition, the Nrf2 signaling pathway is an important protective mechanism for cells against reactive oxygen species (Wu et al., 2017). As expected, our qRT-PCR and western blot results provide evidence that NOT enhanced the expression of Nrf2, Gsr, NQO1, HO-1 and catalase in a dose-dependent fashion. ARE is a downstream factor of Nrf2, which can regulate the expression of antioxidant enzymes (Ishii et al., 2000). Consistently, ARE luciferase activity was also increased by NOT treatment. Taken together, NOT scavenged ROS by increasing the expression of antioxidant enzymes *via* Nrf2/Keap1/ARE pathway.

In order to investigate the effect of NOT on bone loss *in vivo*, we established an OVX mouse model, which is similar to bone changes in postmenopausal women. Micro-CT and H and E staining demonstrated that NOT protected mice against bone loss induced by OVX through improving the number of bone volume, trabecular thickness as well as trabecular number. Consistent with the TRAcP staining *in vitro*, the administration of NOT suppressed TRAcP-positive osteoclasts in the bone. However, *in vivo* experiments we did not observe the role of osteoblasts in the OVX mouse model, which may be another limitation of the study.

**FIGURE 8:**
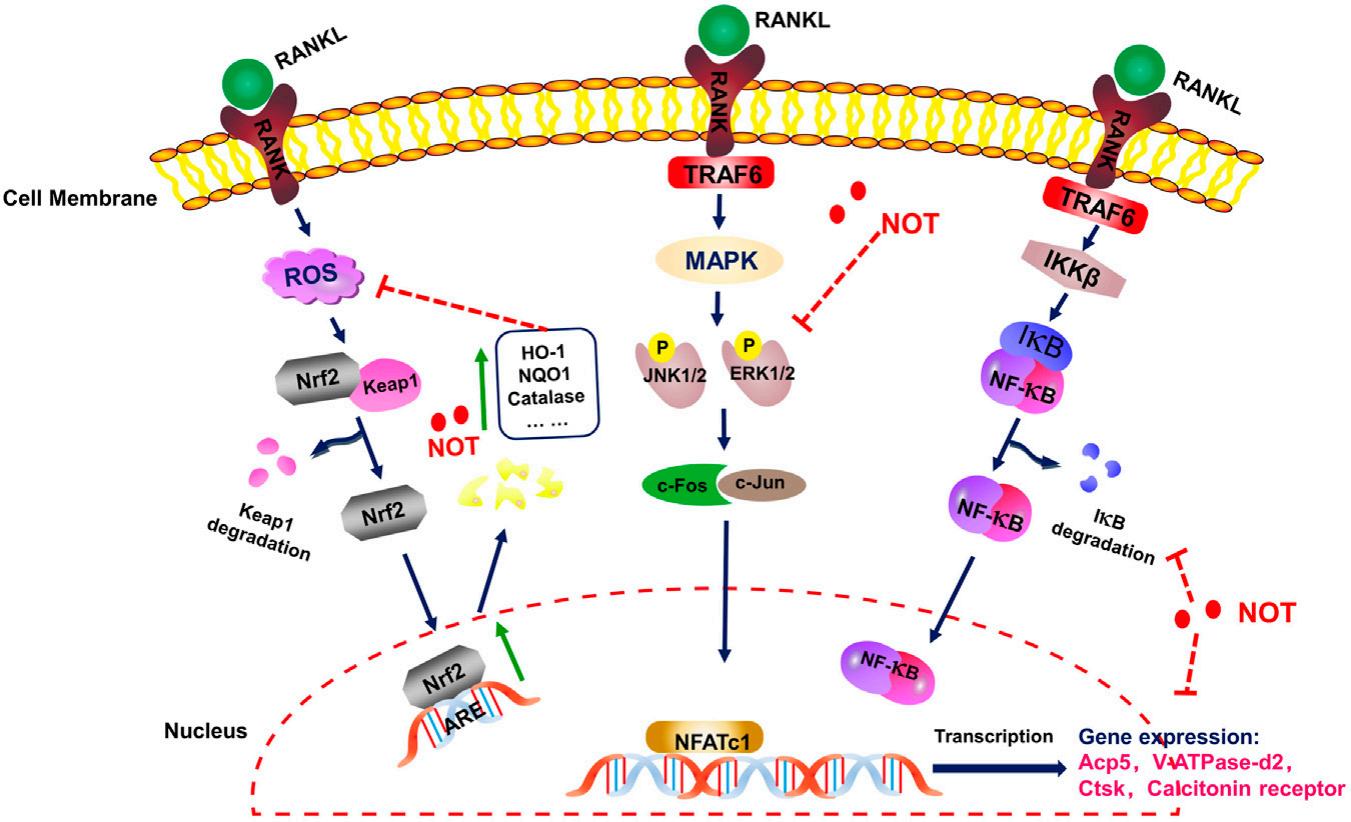
A schematic diagram for illustrating the role of Notopterol in repressing RANKL-mediated osteoclastogenesis through inhibition of NF-κB, MAPK and calcium signaling pathways as well as promotion of ROS scavenging enzymes.

Taken together, we showed for the first time that NOT inhibits RANKL-induced osteoclastogenesis not only by suppressing the MAPK, NF-κB and calcium signaling pathways, but also enhancing the expression of cytoprotective enzymes that scavenge ROS in Nrf2/Keap1/ARE signaling pathways (Figure 8). Consistent with our *in vitro* results, NOT inhibited OVX-mediated bone destruction by repressing the osteoclastogenesis. These findings might provide evidence for the application of NOT in the therapeutic treatment of osteoporosis.

## Data Availability

The original contributions presented in the study are included in the article/Supplementary Material, further inquiries can be directed to the corresponding authors.

## Glossary

Acp5: encoding tartrate resistant acid phosphatase [TRAcP]
ALP: alkaline phosphatase
α-MEM: Alpha modified minimal essential medium
ARE: antioxidant response element
BMMs: bone marrow monocytes
BSA: bovine serum albumin
BV/TV: bone volume/tissue volume
Ct.Ar: cortical bone area
Ct.Ar/Tt.Ar: cortical area fraction
Ct.Th: cortical thickness
Ctsk: encoding cathepsin K
DMSO: dimethyl sulfoxide
EDTA: ethylenediaminetetraacetic
FBS: fetal bovine serum
Gsr: glutathione reductase
H&E: hematoxylin and eosin
HO-1: hemeoxygenase-1
Hprt1: hypoxanthine phosphoribosyltransferase 1
HRT: hormone replacement therapy
Keap1: kelch-like ECH-associated protein-1
MAPK: mitogen-activated protein kinase
M-CSF: macrophage colony-stimulating factor
micro-CT: micro-computed tomography
MMP9: matrix metalloproteinase 9
MTS: tetrazolium salt
NFATc1: nuclear factor of activated T cells 1
NF-κB: nuclear factor-κB
NOT: notopterol
NQO1: NAD(P)H quinine oxidoreductase 1
Nrf2: NF-E2-related factor 2
Oc.S/BS: osteoclast surface/ bone surface
OVX: ovariectomized
PBS: phosphate buffer saline
PFA: paraformaldehyde
PLC: phospholipase C
qRT-PCR: quantitative real-time polymerase chain reaction
RANKL: receptor activator of nuclear factor-κB ligand
RIPA: radioimmune precipitation
ROS: reactive oxygen species
SEM: standard error of mean
SDS-PAGE: sodium dodecyl sulfate-polyacrylamide gel electrophoresis
Tb.Th: trabecular thickness
Tb.Sp: trabecular spacing
TBST: Tris-Buffered Saline and Tween 20
Tb.N: trabecular number
TRAFs: tumor necrosis factor receptor-associated factors
Tt.Ar|: total tissue area
V-ATPase-d2: vacuolar-type H + ATPase V0 subunit d2.
