# Supplementary figures and images for: Notopterol Attenuates Estrogen Deficiency-Induced Osteoporosis via Repressing RANKL Signaling and Reactive Oxygen Species
Source: Front Pharmacol. 2021 Jun 3;12:664836. doi: 10.3389/fphar.2021.664836 (PMC8210423; doi:10.3389/fphar.2021.664836)

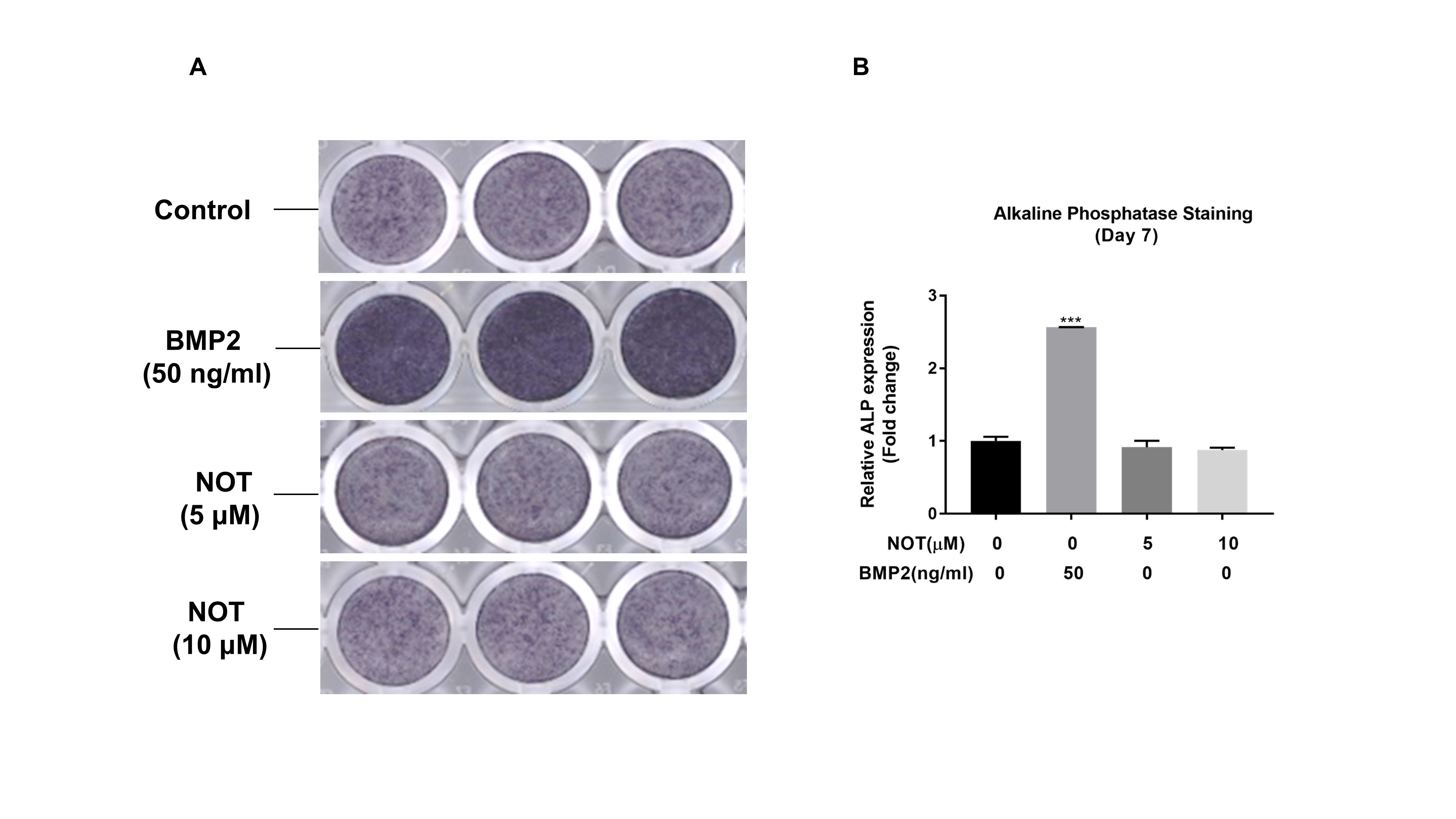

Supplement: Supplementary file 1 [file Image3.JPEG]

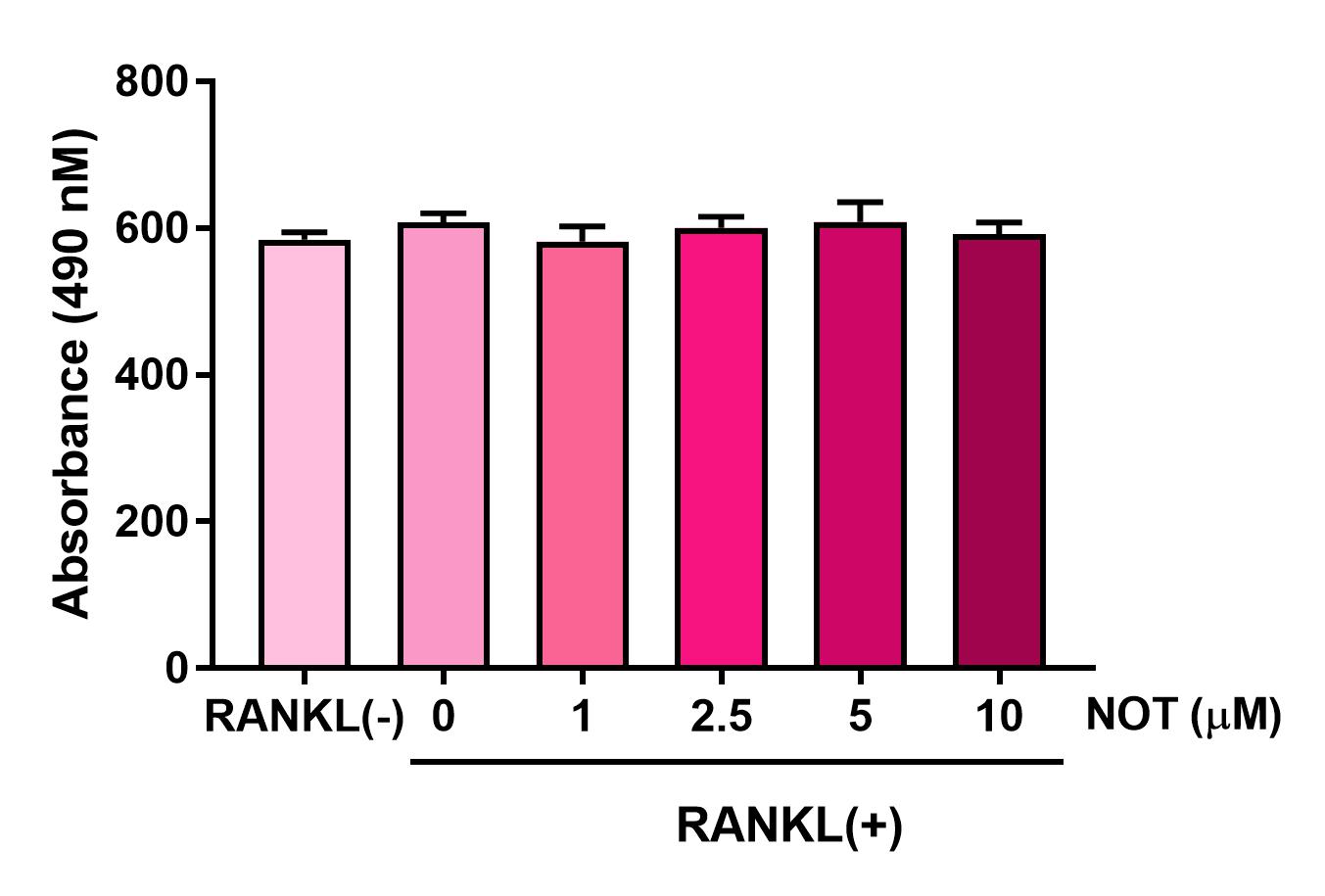

Supplement: Supplementary file 2 [file Image1.JPEG]

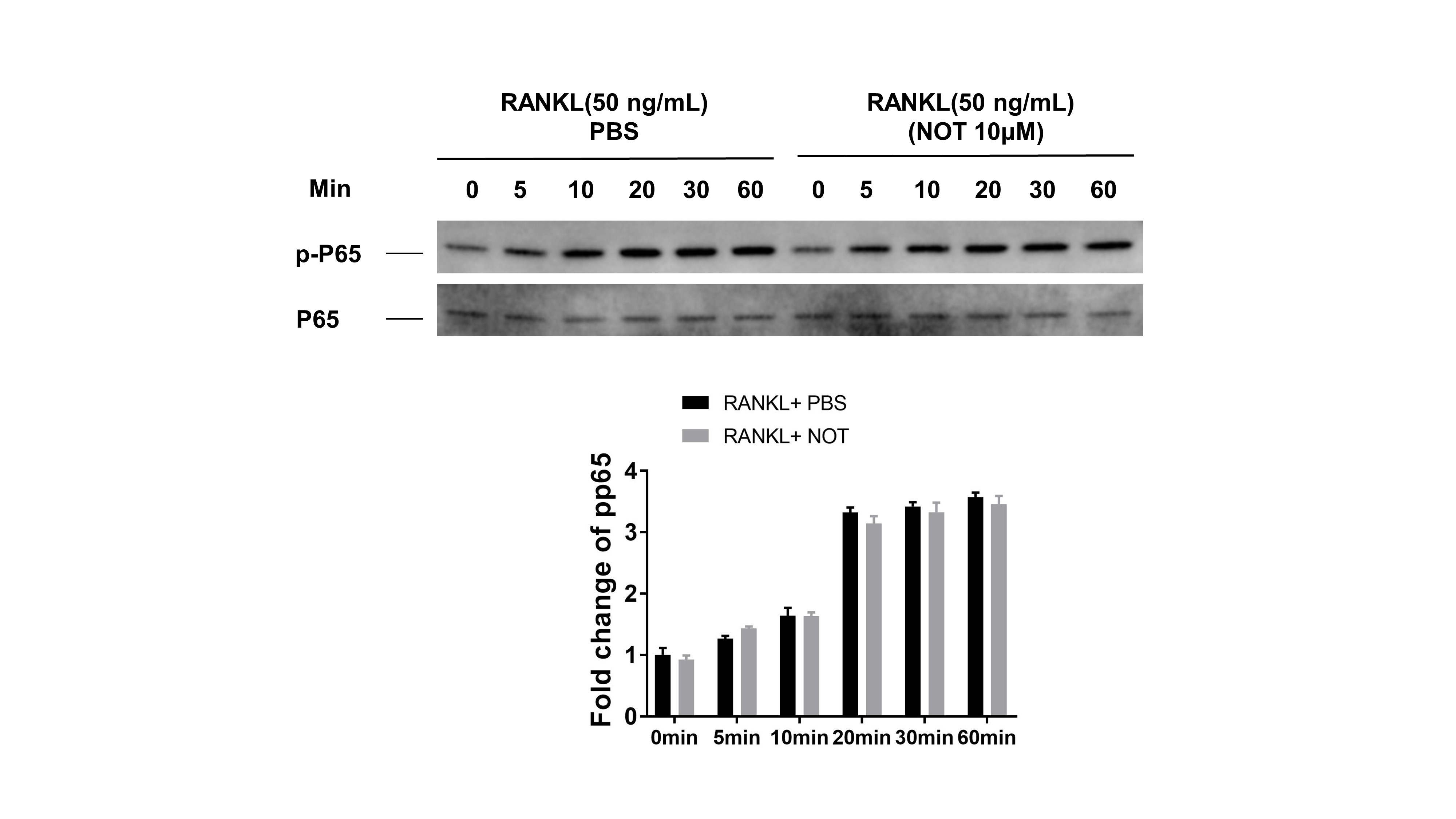

Supplement: Supplementary file 3 [file Image4.JPEG]

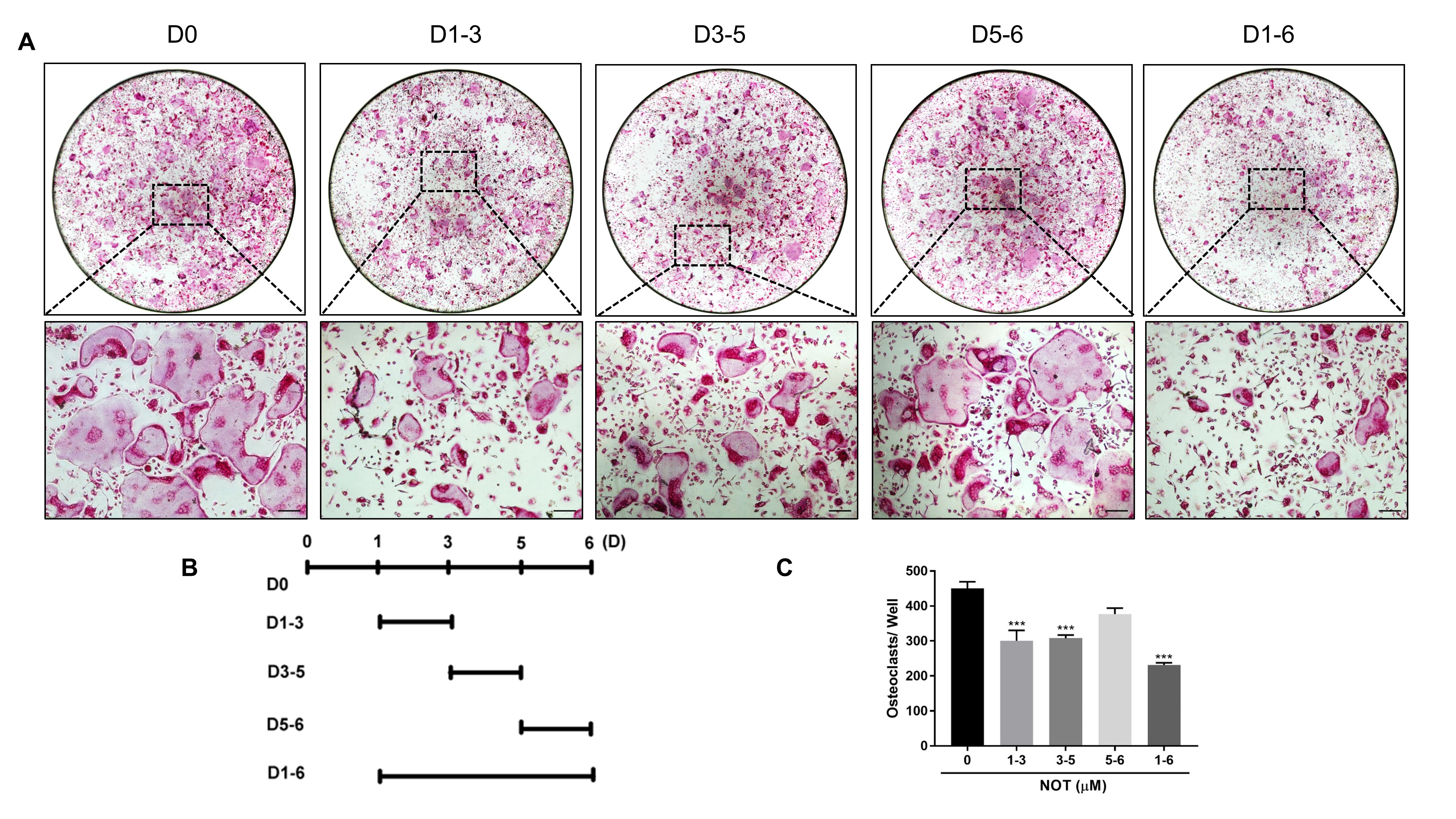

Supplement: Supplementary file 4 [file Image2.JPEG]

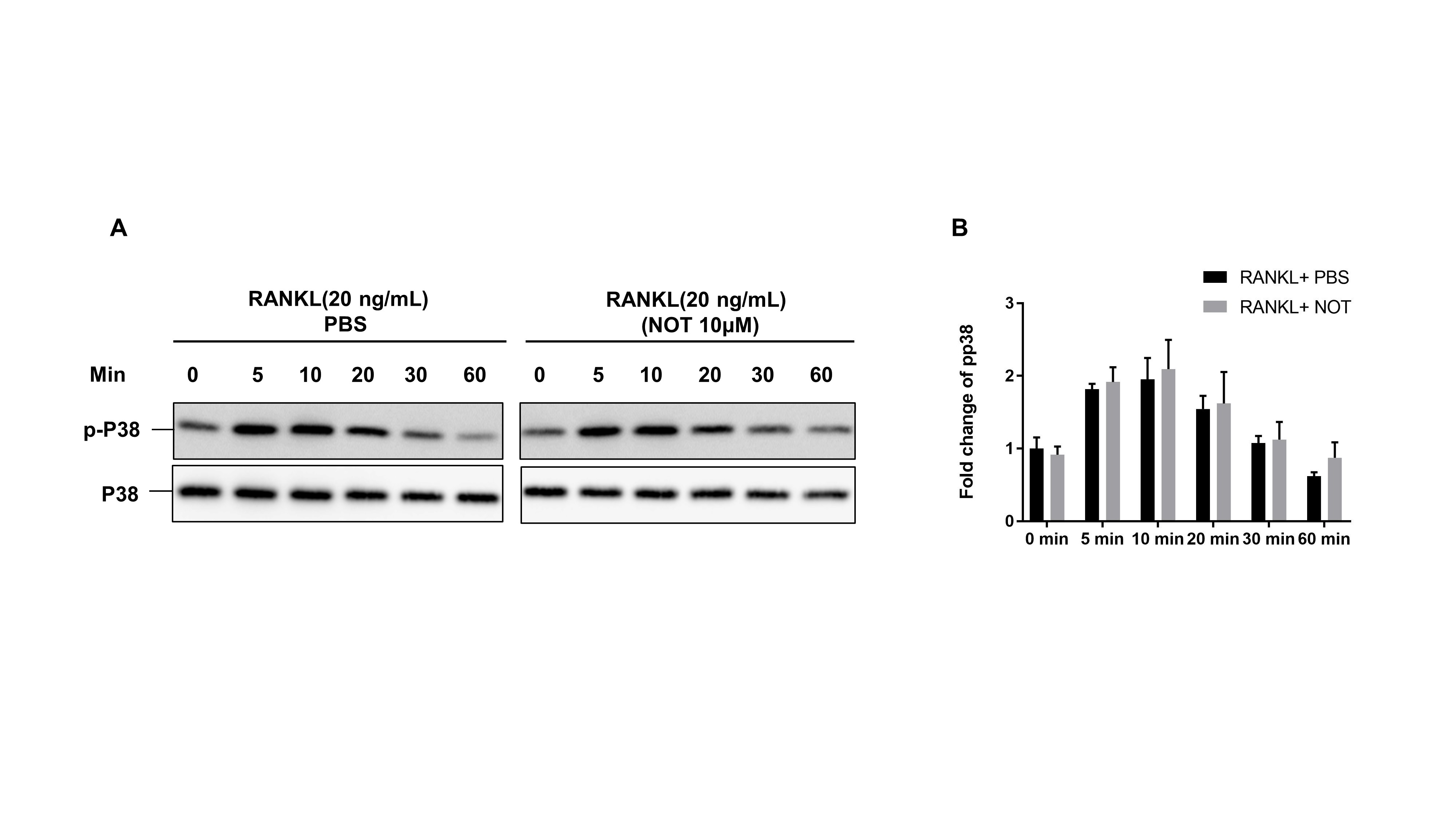

Supplement: Supplementary file 5 [file Image5.JPEG]

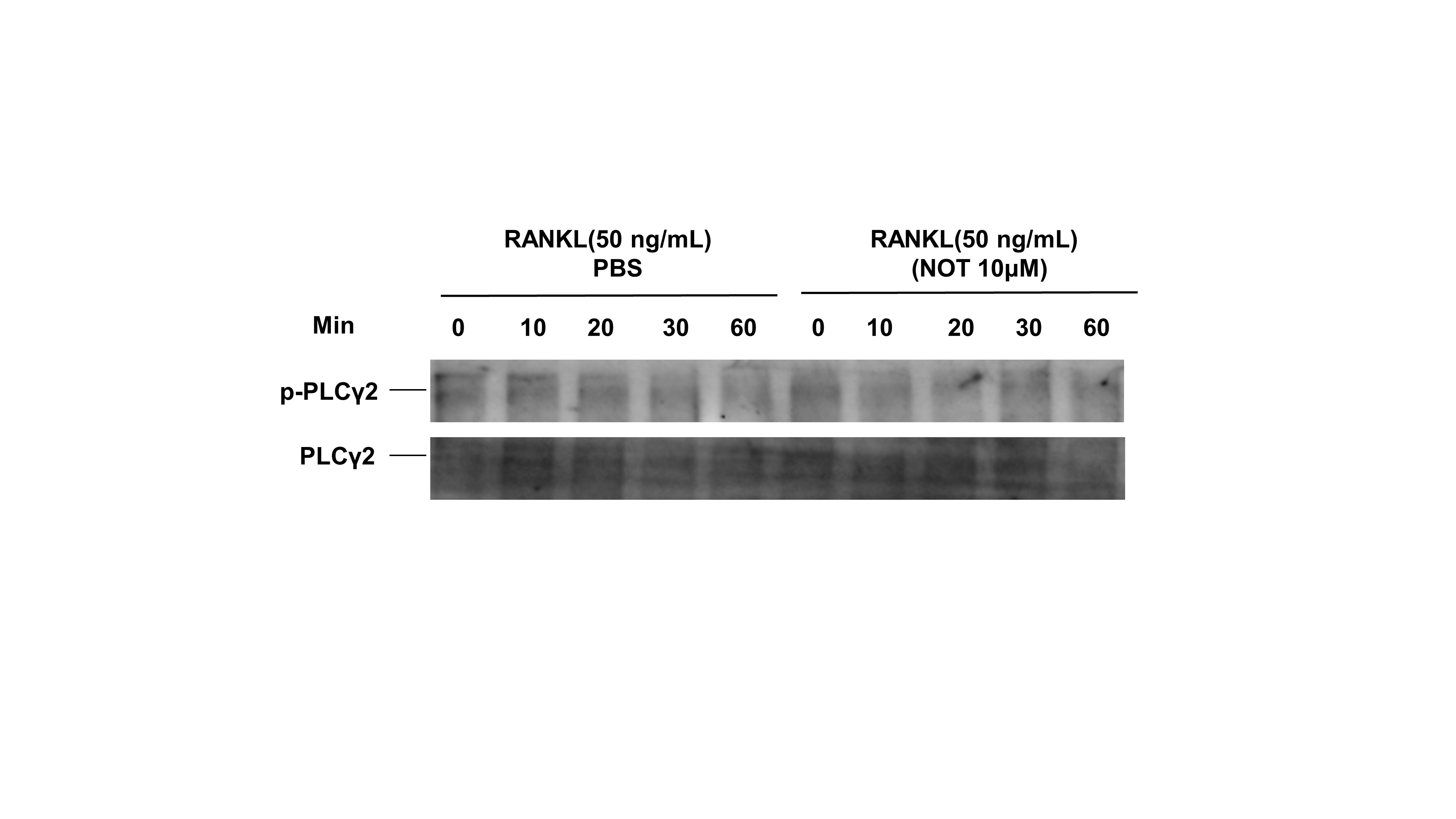

Supplement: Supplementary file 6 [file Image6.JPEG]
